# Taste Preferences at Different Ambient Temperatures and Associated Changes in Gut Microbiota and Body Weight in Mice

**DOI:** 10.3390/foods13132121

**Published:** 2024-07-03

**Authors:** Xing Zhang, Hui He, Tao Hou

**Affiliations:** 1 College of Food Science and Technology, Huazhong Agricultural University, Wuhan 430070, China; zhangxing@nbu.edu.cn (X.Z.); hehui@mail.hzau.edu.cn (H.H.); 2 Institute of Drug Discovery and Technology, Ningbo University, Ningbo 315211, China; 3 Key Laboratory of Environment Correlative Dietology, Huazhong Agricultural University, Ministry of Education, Wuhan 430070, China

**Keywords:** food flavors, ambient temperature, gut microbiota, metabolic pathways

## Abstract

Taste, dietary choices, and gut microbiota are often analyzed as major factors of metabolic health. Populations living in cold or hot regions have different dietary habits. This study aims to investigate the potential association among ambient temperature, food taste preferences, and cecal microbiota community profiles in mice. By exposing mice to mixed diets containing sweet, sour, salty, and bitter flavors at low (4 °C) and high (37 °C) ambient temperatures, the taste preferences of mice at both ambient temperatures were in the order of saltiness > sweetness > bitterness > sourness. Exposing mice to sweet, sour, salty, and bitter diets, respectively, revealed that in a low-temperature environment, mice consuming salty (5.00 ± 1.49 g), sweet (4.99 ± 0.35 g), and sour (3.90 ± 0.61 g) diets had significantly higher weight gain compared to those consuming normal feeds (2.34 ± 0.43 g, *p* < 0.05). Conversely, in a high-temperature environment, no significant changes in body weight were observed among mice consuming different flavored diets (*p* > 0.05). In a low-temperature environment, mice fed sour and sweet diets showed a significant difference in the gut microbiota composition when compared to those fed a normal diet. A higher abundance of *Lachnospiraceae*, *UBA1819*, and *Clostridiales* was identified as the most significant taxa in the sour group, and a higher abundance of *Ruminiclostridium* was identified in the sweet group. These differences were associated with microbial pathways involved in carbohydrate metabolism, amino acid metabolism, and energy metabolism. A high-temperature environment exhibited only minor effects on the gut microbiota profile. Overall, our findings provide evidence for temperature-modulated responses to the taste, gut microbiota functions, and body weight changes in mice.

## 1. Introduction

Food taste preferences and cravings have been found to be associated with living environments. Diet choices are predominantly guided by food flavors, with sweet, sour, salty, and bitter recognized as primary tastes [1]. Research indicates that temperature plays a significant role in the sensory features of these tastes. Colder environments tend to intensify bitter tastes, while warmer environments enhance fruit flavors [2]. These temperature-induced taste variations can alter food preferences and dietary habits, impacting nutritional characteristics and health status. For instance, prolonged intake of sweets leads to decreased impulse control and a lower ability to resist the craving for sweets [3] and increases the risk for metabolic and neurodegenerative diseases [4]. Similarly, enhanced preferences for salty tastes induce the attendant risk of hypertension [5]. Other tastes such as umami or bitter have also been associated with alterations in blood pressure [5]. In addition, the consumption of acidic substances such as vinegar could modulate hyperlipidemia by reducing serum cholesterol and triacylglycerols [6]. These results indicate a significant association between food cravings and metabolic disease.

Diet has emerged as a main contributor to the microbiota composition and functional capacity. The gut microbial communities, including the structure and abundance of the flora, have been suggested as a predictor of the metabolic health of the host [7]. The microorganisms hosted by the gastrointestinal tract are involved in numerous physiological and metabolic processes including modulation of the appetite and the regulation of energy in the host. For instance, studies suggest that changes in energy harvesting in microbiota are associated with obesity, and the composition of these energy harvesting groups may be controlled by taste receptors [8]. Intestinal bacteria may play a role in determining dietary preferences by regulating the expression of taste receptors. Bitter, sweet, and umami taste receptors in the gut are activated by certain nutrients, which consequently regulate appetite and body weight and maintain homeostasis by targeting hormone secretion or regulating the intestinal flora [9]. Intestinal bacteria also play a significant role in influencing taste preferences by interacting with the host’s chemosensory signaling pathways, affecting both short- and long-term ingestive behavior [10]. The gut microbiota produces metabolites and stimulates the synthesis of hormones and neurotransmitters that can impact the host’s eating behavior, including the expression of taste receptors and the modulation of taste sensations [11]. Moreover, dysbiosis in the intestinal microbiota, often observed in pathological conditions, may further contribute to changes in taste preference and food intake, highlighting the crucial role of gut microbes in shaping taste habits and preferences [12]. Therefore, the gut microbial community may play an indispensable role in metabolic disease induced by taste preference. However, the associations between food flavors, the gut microbial community, and the metabolic mechanisms responsible are yet to be fully elucidated.

In this work, we hypothesize that ambient temperature may influence food preferences and consequently result in metabolic changes, such as alterations in body weight and metabolic pathways. Changes in the structure of the gut may respond to physical changes caused by different flavors of food. Two experiments were performed to investigate the food preferences of mice in different temperature environments and the effects of different flavors of food on changes in the body and intestinal flora of mice. We aim to provide a basis for the study of the interaction between dietary behavior and gut health.

## 2. Materials and Methods

### 2.1. Animal Design

Adult male C57BL/6J mice (20 g ± 1 g) were obtained from the Laboratory Animals of Huazhong Agricultural University and maintained in an alternating 12 h light and dark cycle system with a humidity of 50 ± 10%. Animal procedures were performed according to the Guidelines for the Care and Use of Laboratory Animals of Huazhong Agricultural University and the animal ethics approval number was HZAUMO-2020-0073. 

Experiment I:

Mice were subjected to two artificial climate incubators (Jiangnan instrument Co., Ltd., Ningbo, China). One low temperature (4 °C) and one high temperature (37 °C) were set inside the incubators, respectively. Thirty mice were subjected to two artificial climate incubators with low and high temperatures inside, respectively, and acclimated for one week. Then, mice kept at each temperature were fed a measured dose of forage with different flavors (sweet, sour, bitter, and salty). Table 1 shows the formulation of the different flavors of feed. The animals were free to choose and consume the different flavors of feed during the 30-day housing. Six parallel experimental groups (5 mice per group) were set up at each temperature. The consumption of each flavor was recorded every other day. After 12 days of feeding, the most popular flavor was removed and the mice’s flavor choice continued to be observed. On day 22, the most popular flavor of the three remaining flavors was removed, and the consumption of the two remaining flavors continued to be observed in the following days. Taste preferences were assessed by comparing the quantities of each flavor consumed during each phase of the experiment. The flavor consumed in the greatest quantity during each observation period was considered the most preferred. This stepwise removal of flavors allowed for a detailed analysis of how temperature influences changes in taste preference over time.

Experiment II:

Twenty-five mice were housed at each temperature. After one-week acclimation, the mice were divided into 5 groups (5 mice per experimental group) in each incubator and obtained normal, sweet, sour, bitter, and salty flavors of feed, respectively. The stimuli and stimulus concentrations are referenced in the study by Bo et al. [13]. The body weight of the mice in each group was monitored every other day. By tracking the body weight every other day, we observed the trends in the weight changes corresponding to the consumption of different flavored feeds. After 4 weeks of feeding, the mice were fasted for 12 h and sacrificed by cervical dislocation. Cecum samples were collected in liquid nitrogen for gut microbiome analysis.

### 2.2. Gut Microbiota Analysis

The bacterial DNA from the mice caecum was extracted using a TGuide S96 magnetic soil and stool DNA kit (Tiangen Biotech Co., Ltd., Beijing, China) according to the instructions of the manufacturer. The V3-V4 region of 16S rRNA genes was amplified through polymerase chain reaction (PCR) using the forward primer 338 F (5-ACTCCTACGGGAGGCAGCA-3) and the reverse primer 806 R (5-GGACTACHVGGGTWCTAAT-3). The PCR reaction was run according to the following protocol: pre-denaturation at 95 °C for 3 min, 25 cycles of denaturing at 95 °C for 30 s, annealing at 50 °C for 30 s, elongation at 72 °C for 45 s, and a final annealing extension step at 72 °C for 7 min. The amplicons were purified using an E.Z.N.A.^®^ Gel Extraction Kit (Omega Bio-Tek, Norcross, GA, USA) and quantified using QuantiFluor TM-ST (Promega, Madison, WI, USA). The purified amplicons were sequenced on the Illumina novaseq6000 platform (Illumina, San Diego, CA, USA) by Biomarker Technologies Co., Ltd. (Beijing, China). The read sequences were clustered into OTUs with a 97% similarity cutoff using Vsearch software 2.8.1 [14]. The representative read of each OTU was selected using the QIIME package. All the representative reads were annotated and blasted against the Silva database Version 123 using the RDP classifier (confidence threshold was 70%).

### 2.3. Beta Diversity Analysis

The Beta (β) diversity was calculated using a Canberra distance matrix, which is a non-phylogenetic, equal-weight measure where each OTU affects the distance value equally.

### 2.4. Predicted Metagenome

Metagenomic predictions were made using Phylogenetic Investigation of Communities by Reconstruction of Unobserved States (PICRUSt) and summarized as KEGG (Kyoto Encyclopaedia of Genes and Genomes) pathways [15]. The functional information of the top 15 in the maximum abundance of each group was selected.

### 2.5. Statistical Analysis

All the data are presented as the mean value ± standard deviation (SD). Statistical significance was determined using one-way analysis of variance (ANOVA) through SPSS 21, and *p* < 0.05 was considered statistically significant.

## 3. Results

### 3.1. Taste Preference and Weight Changes

**Figure 1:**
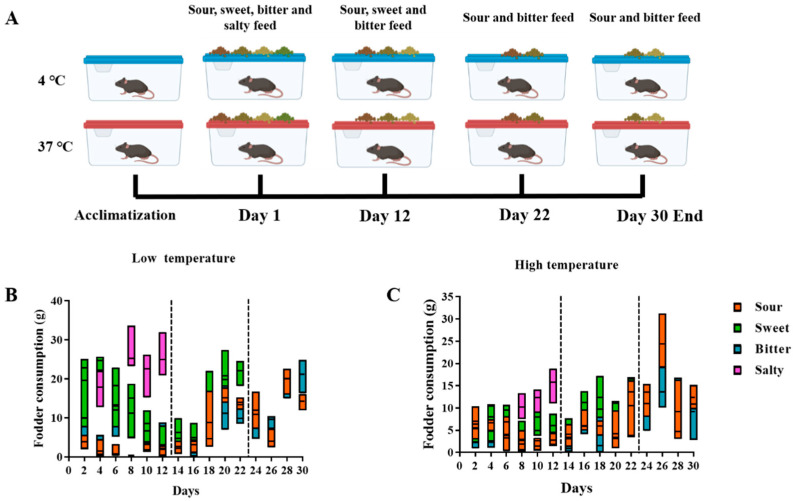
Consumption of different flavors of feed in Experiment I, where mice were allowed to freely choose different flavors of feed. (**A**) The overall flow of Experiment I. The mice were initially provided with sour, sweet, bitter, and salty feed for 12 days. Then, the most consumed flavor was removed and the consumption of the remaining three flavors of feed was observed for a further 10 days. At day 22, the most popular flavor of the remaining feed was removed and observed for a further 8 days. (**B**,**C**) Consumption of different flavors of feed by mice at low (**B**) and high (**C**) temperatures.

Food consumption and taste preference within sweet, sour, bitter, and salty feedings were monitored among the mice kept at cold and high temperatures (Experiment I, Figure 1A). The results showed that the mice preferred salty and sweet flavors during the first 12 days. Following the removal of salty feed, sweet became the most popular flavor over the next 10 days compared to sour and bitter (Day 13–Day 22). After removing the sweet feed, the mice living in a high-temperature environment preferred the sour taste to the bitter taste, whereas the preference for sweet and sour flavors varied between the mice living in a cold-temperature environment in the last week (Day 23–Day 30, Figure 1B,C).

**Figure 2:**
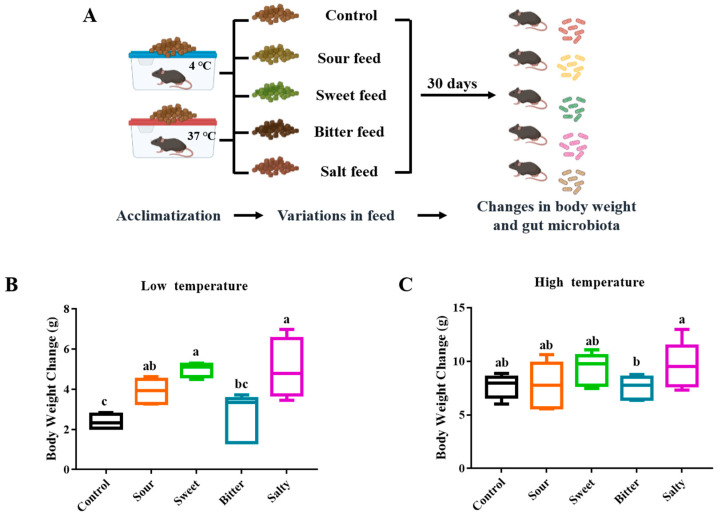
**Body weight changes of mice fed with normal, sour, sweet, bitter, and salty forages in Experiment II.** (**A**) The overall flow of Experiment II. The mice were provided with normal, sour, sweet, bitter, and salty feed, respectively, for 30 days in low and high temperatures. (**B**,**C**) Body weight changes of mice fed with normal, sour, sweet, bitter, and salty forages at low (**B**) and high (**C**) temperatures. The column marked with ^a, b, c^ indicates a significant difference from the other groups (*p* < 0.05).

To study the effect of different food tastes on the body weight of mice, sweet, sour, bitter and salty tastes were provided to mice in low- and high-temperature environments, respectively (Experiment II, Figure 2A). As shown in Figure 2B,C, over the 4-week feeding period, the mice tested with salty (5.00 ± 1.49 g), sweet (4.99 ± 0.35 g), and sour (3.90 ± 0.61 g) flavors showed a significant increase in body weight gain compared to the normal group (2.34 ± 0.43 g, *p* < 0.05) in the low-temperature environment, while there was no significant difference in the body weight changes between the mice consuming bitter and normal feed (Figure 2B, *p* > 0.05). In addition, no significant differences were observed between mice fed with various flavors of diets in a high-temperature environment (Figure 2C, *p* > 0.05).

### 3.2. Changes in Gut Microbiota Structure Associated with Food Flavors

**Figure 3:**
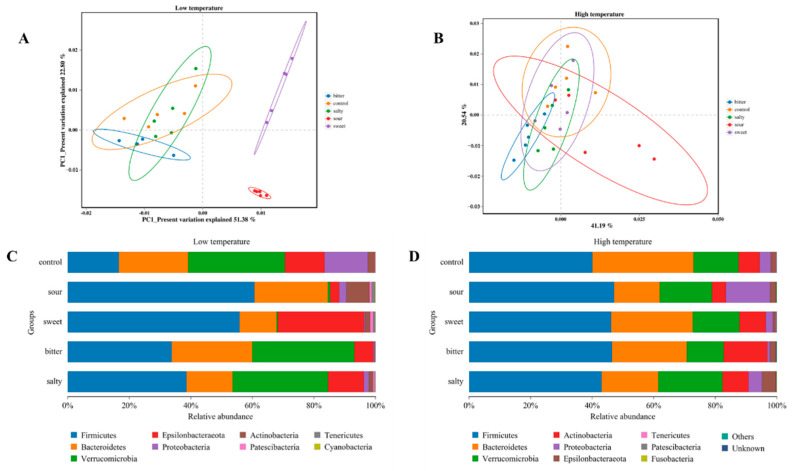
**Changes in intestinal microbial abundance in mice after ingestion of different flavored diets.** (**A**,**B**) Analysis of β diversity at the level of the operational taxonomic unit at low and high temperatures. (**C**,**D**) Composition of gut microbiota at different phylum levels at low and high temperatures.

To investigate the effect of different food tastes on the structure of the gut microbiota, we analyzed the β-diversity of the intestinal microbiota in mice consuming different flavored diets at high- and low-ambient temperatures. As shown in Figure 3A, at the low-temperature condition, the samples collected from the sweet and sour diet groups showed a significantly different microbiota structure compared to the control, bitter, and salty diet groups. A significant difference in the microbiota diversity was also observed between the sweet and sour groups. However, there was no significant difference in the β-diversity of the gut microbiota among the control, sweet, sour, bitter, and salt diet groups in mice exposed to high temperatures, suggesting that ambient temperature is an essential influence on intestinal flora (Figure 3B).

The relative abundances of gut microbiota at the phylum level were identified. In a low-temperature environment, sweet, sour, bitter, and salt diets induced a higher abundance of *Firmicutes* compared with the control group (*p* < 0.05). The abundance of *Bacteroides* was significantly lower in the sweet and salty groups, and the abundance of *Verrucomicrobia* was significantly lower in the sour and sweet groups in comparison with the control group (Figure 3C, *p* < 0.05). In the high-temperature environment, the abundance of *Proteobacteria* was higher and *Bacteroidetes* and *Anctinobacteria* were lower in the sour diet group compared to the control group (Figure 3D, *p* < 0.05). The salty diet demonstrated a higher abundance of *Verrucomicrobia* and a lower abundance of Bacteroides (*p* < 0.05). However, the sweet diet showed a minor impact on the microbiota composition in vivo and did not affect bacterial diversity at the phylum level.

**Figure 4:**
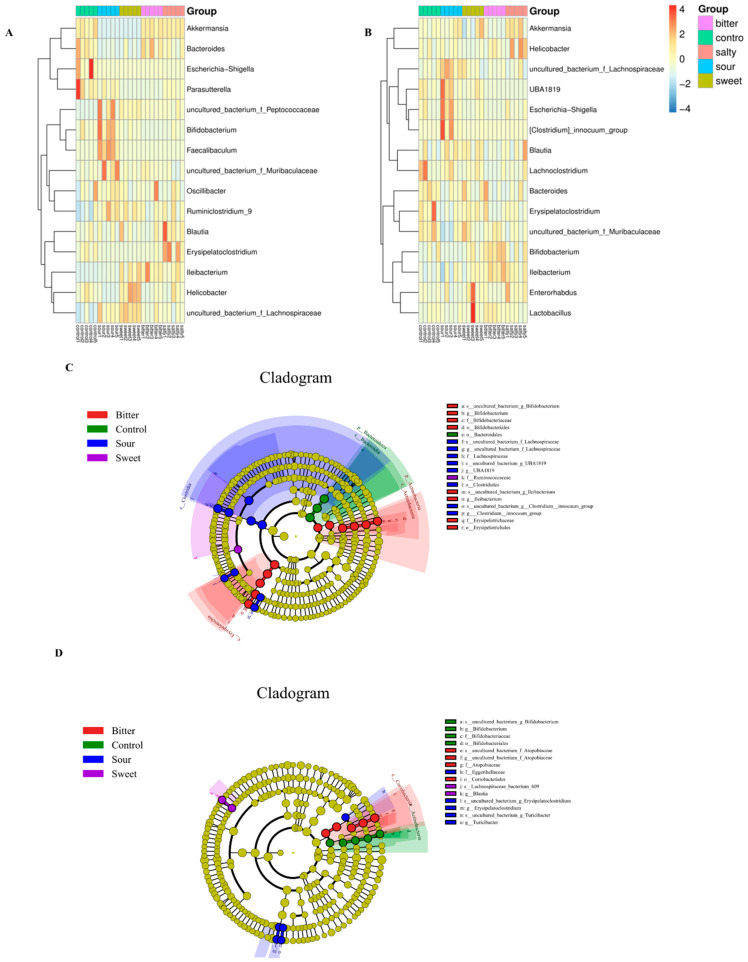
**Taxonomy comparison of the gut microbiome and identification of biomarker’s taxa at the genus level.** The relative abundance of the top 15 gut bacterial genera in mice fed normal, sour, sweet, bitter, and salty diets at low (**A**) and high (**B**) temperatures. The taxonomic cladogram was obtained through linear discriminant analysis effect size analysis (LEfSe). LefSe determined that 15 flora at the genus level were markedly enriched in the normal, sour, sweet, bitter, and salty diet groups at low (**C**) and high (**D**) temperatures. The diameter of each circle is proportional to the abundance of the taxon. The size of each circle is directly proportional to the abundance of the corresponding taxonomic unit.

We then determined the effect of different flavors on the changes in the microbiota structure at the gene level. As shown in Figure 4A,B, during 4 °C acclimation, the relative abundance of *Akkermasnsia*, *Bacteroides*, and *Helicobacter* was reduced and *Bifidobacterium*, *Faecalibaculum*, *Ruminiclostridium_9*, and *Lachnospiraceae* were increased in the sour diet group (*p* < 0.05). However, there was no significant difference in these changes in the sour diet mice in a 37 °C housing environment. Similarly, a reduction in *Akkermasnsia* and *Bacteroides* and an elevation in *Helicobacter*, *Ruminiclostridium_9*, and *Lachnospiraceae* abundance with the sweet feed were observed only at a low ambient temperature (*p* < 0.05). In particular, the level of *Ileibacterium*, which is reported to be associated with energy expenditure and may protect against obesity in germ-free mice [16], was increased by bitter feed in both the 4 °C and 37 °C housing.

### 3.3. The Correlations of Ambient Temperature, Tastes, and Bacterial Taxa

The linear discriminant analysis (LDA) effect size (LefSe) was used to illustrate the most significantly differentially abundant taxa within the microbiota with different diet groups. As shown in Figure 4C,D, the bitter, sour, and sweet diet groups yielded the most distinctive microorganisms. At a low temperature, LEfSe revealed that the genera *Bifidobacterium*, *Ileibacterium*, and *Erysipelotrichaceae* were the most abundant in the bitter group. A higher abundance of *Lachnospiraceae*, *UBA1819*, and *Clostridiales* was observed in the sour group and a higher abundance of *Ruminiclostridium* in the sweet group (Figure 4C).

In the high-temperature groups, higher enrichment of *Atopobiaceae* and *Coriobacteriales* was associated with the bitter diet group, and higher enrichment of Eggerthellaceae, *Erysipelatoclostridium*, and *Turicibacter* was associated with the sour diet group. In addition, the sweet diet group was characterized by *Lachnospiraceae* and *Blautia*, while the salty diet group in the 4 °C and 37 °C accommodation was not significantly associated with the abundance of any bacterial taxa according to the LEfSe analysis (Figure 4D).

### 3.4. Functional Diversity among the Different Taste Treatments under Different Temperature Conditions

**Figure 5:**
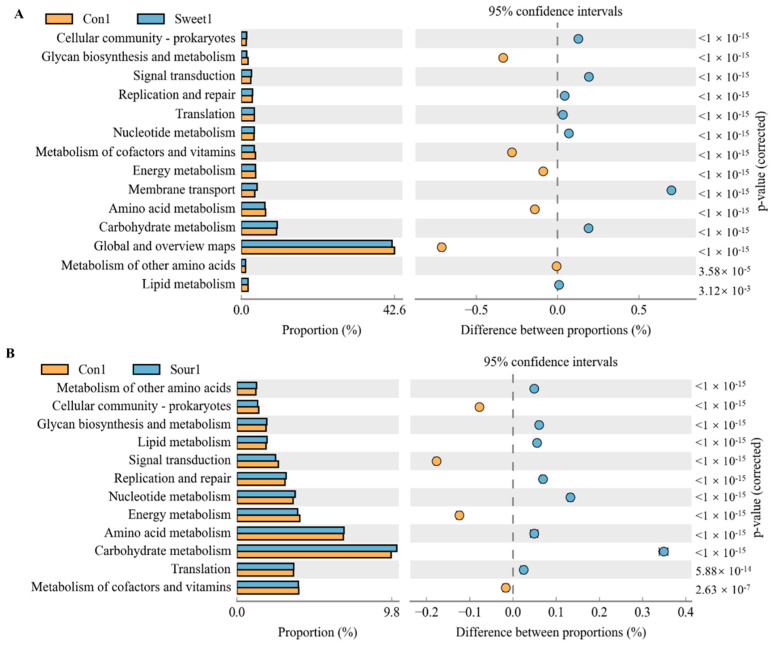
**Significant KEGG pathways at level 2 for the gut microbiota of mice after ingestion of different flavored diets at low temperatures.** (**A**) Control group vs. sweet group. (**B**) Control group vs. sour group. Differences in abundances between the control and sweet and sour diet groups were compared using a *t*-test in STAMP, *p* < 0.05.

To predict the potential links between changes in gut macrobacteria and taste responsiveness, as well as the possibility that bacterial metabolism may modulate or enhance the functional category, we further analyzed the correlations between bacterial taxa and metabolic phenotypes in the KEGG pathway using PICRUSt analyses. According to KEGG level 2, at a low temperature, signal transduction, replication and repair, membrane transport, and carbohydrate metabolism were the most dominant categories in the sweet feed group in comparison with the control group (Figure 5A,B, *p* < 0.05). The sweet feed was also negatively related to glycan biosynthesis and metabolism, metabolic cofactors and vitamins and amino acid metabolism pathways. In addition, the sour feed was positively related to glycan biosynthesis and metabolism, lipid metabolism, replication repair, nucleotide metabolism, and carbohydrate metabolism and negatively related to signal transduction and energy metabolic pathways. However, no special functional prediction results were shown among the normal, sweet, sour, bitter, and salty taste groups at high temperatures.

## 4. Discussion

### 4.1. Dietary Preferences and Weight Change

Diet and ambient temperature have a major impact on health and quality of life. Taste is one of the most important factors when people make choices for food. This study investigated how significantly environmental temperatures influence taste choices. Our results show that at both low and high temperatures, mice provided with an alternative diet of sweet, sour, bitter, and salty tastes were found to have the strongest preference for sweet and salty flavors (Figure 1B,C). A previous study reported that the consumption of sweet and salty diets is predisposed by evolutionarily driven taste preferences. Both sweet and salty tastes are associated with strong hedonic appeal [17]. The ability to detect sweet tastes interacts with the systems controlling the effect. Natural, sweet-tasting substances induce the hedonically positive and highly motivating sensory quality. They also serve as sources of available energy [18]. Salty preference is likely to be shaped by innate components. Studies suggest that early experiences, both in utero and during infancy, may shape the preference for salty taste [19]. Sweetness attracts people to breast milk and fruit and saltiness attracts them to sodium and possibly other minerals needed for bone growth. These preferences have evolved into a desire to consume sweet sources of energy and salty minerals during periods of growth [20]. This may explain the finding that salty and sweet flavors are the two most preferred tastes of mice in both high- and low-temperature environments.

Dietary compounds may stimulate taste receptors throughout the digestive tract, which potentially affect taste receptor expression, digestive tract function, and the digestive tract microbiota, thereby affecting weight and health [8]. Body weight gain could be an indication of the increase in adiposity and bone growth associated with sweet and salt preferences. Sweet foods can affect body weight through various mechanisms. Sweet foods are often calorie-dense, leading to increased caloric intake and potential weight gain if not balanced by energy expenditure [21]. The rewarding properties of sugar can stimulate brain reward pathways, increasing cravings and the consumption of sweet foods, contributing to weight gain [22]. Additionally, high sugar intake can cause metabolic disturbances such as insulin resistance and increased fat accumulation, impacting body weight regulation [22]. Salty taste has been reported to be closely related to body fat. A high salt diet has been linked to weight gain and adiposity in juvenile female rats [23]. Research showed that a moderate short-term increase in salt intake decreased diet-induced thermogenesis, which could contribute to weight gain in populations consuming a Western diet high in salt [24]. The development of the human hedonic response to salt may be attributed to the stimulation of opiate and dopamine receptors in the brain’s reward and pleasure center. This addiction subsequently increases calorie consumption and augments the incidence of overeating, overweight, obesity, and related illnesses. Thus, obesity and related diseases may be symptoms of salted food addiction [25].

Although studies have shown that a cold stimulus could increase taste sensitivity [26], this enhancement seems to not affect taste preference. Our results suggest that the taste preferences of mice follow the same trend in both low- and high-temperature environments. Previous research suggests that odor, taste, or hunger ratings were not affected by short-term exposure to changes in ambient temperature [27]. Therefore, it was reasonable that the taste preference did not change in the cold or hot environments, while significant differences in body weight were observed among the groups of mice consuming feeds with different flavors in low-temperature environments rather than high-temperature environments. A study on the food cravings of firefighters in hot conditions found that high-temperature restriction did not impact the subjective ratings of hunger and cravings for food, but the timing of the food intake differed under hot conditions [28]. The regulation of feeding times and the lower duration of non-feeding activities under hot exposure may have led to non-significant changes in body weight in mice [29].

### 4.2. Impact of Different Tastes on the Function of Gut Microbiota

The gut microbiota plays a significant role in taste perception, appetite regulation, and metabolic processes [30]. In this study, the consumption of different flavors showed different microbiological profiles in the cecum of mice. At a low temperature, the β diversity of sweet and sour diets showed significant differences from that of normal diets. Some acidic substances, such as organic acids, can maintain the integrity of the cellular gut barrier, modulate intestinal microbiota, and improve digestion and nutrient absorption rates [31]. There was evidence of reduced pH in the cecal content of chicks supplemented with 5 g/kg dietary citric acid [32]. These results suggest that changes in bacterial levels may have been due to the lower cecum pH, as a lower pH is conducive to the growth of beneficial bacteria and limits the growth of pathogenic bacteria, which require a relatively higher pH to grow [33]. Our results showed that higher abundances of *Lachnospiraceae* were found in the sour group in low- and high-temperature conditions; LEfSe analysis confirmed the same taxa to be significant as well. *Lachnospiraceae* are known for their probiotic functions, fermentation capabilities, and production of metabolites like short-chain fatty acids (SCFAs) [34]. These bacteria can modulate intestinal permeability and impact host lipid metabolism pathways, making them a potential adjunctive treatment for obesity [34]. This modulation is reflected in the functional pathways of carbohydrate metabolism (Figure 5B).

The consumption of large amounts of carbohydrates such as sucrose and high fructose corn syrup is associated with profound changes in the gut microbiota, and the associated dysbiosis of the gut microbiota is partly responsible for the development of obesity [35]. Our results suggested that the sweet feed induced an increased *Firmicutes* to *Bacteroides* ratio [36]. The same result was also observed in the salty group. The balance between *Firmicutes* and *Bacteroidetes* has been associated with obesity and metabolic health. An increased ratio of *Firmicutes* to *Bacteroidetes* has been observed in individuals with obesity, and this imbalance may be influenced by dietary factors [37]. Since mice have a strong preference for sweet and salty feeds at low temperatures, this preference may cause changes in energy metabolism and corresponding changes in the intestinal microflora involved in the regulation of energy metabolism [8]. At the genus level, *Akkermansia*, *Helicobacter*, *Bifidobacterium*, *Lachnospiraceae*, and *Bacteroides* are more influenced by taste variation (Figure 3A,B). *Bacteroides* and *Akkermansia* have been associated with significant effects in improving inflammatory status and immunity and preventing diabetes and obesity [38]. Unfortunately, the sweet and salty diet resulted in significantly lower *Akkermansia* and *Bacteroides* abundance in low-temperature conditions. *Akkermansia* plays a crucial role in regulating body weight and metabolism by influencing various metabolic disorders [39]. Studies on mice have shown that *Akkermansia* can alleviate weight gain, hepatic steatosis, and liver injury induced by a high-fat diet [40]. Similarly, *Bacteroides* species are important for regulating body weight and metabolism through various mechanisms. They have been found to reduce body weight gain, plasma cholesterol, triglycerides, and glucose levels in obese mice while modulating the gut microbiota and immune responses associated with obesity [41]. These changes in the gut microbiome, mediated by a sweet and salty diet, are key reasons for the differential effects on host health and metabolism, as evidenced by weight gain in mice reared in low-temperature environments.

In combination with the body weight gain, our results suggested that the changes in the gut microbiota caused by sour and sweet diets were actively involved in the metabolism process. Some specific functional pathways were correlated negatively with the sweet and sour taste during 4 °C acclimation (Figure 5). In a functional classification based on the KEGG, signaling pathways involved in carbohydrate metabolism, amino acid metabolism, and energy metabolism showed high levels of enrichment in taste changes. These results imply that the microbial community is critical for the metabolic and cellular process as well as the ameliorative effect of a variety of flavors on the microbial community and body health. In addition, our results suggested that the four flavors of the sweet, sour, bitter, and salty diets caused significantly higher differences in the body weight and intestinal flora in mice at low temperatures than those at high temperatures. This could be related to the sensitivity of the taste receptors. For example, sensitivity to sucrose and quinine is increased by warming from 10 °C, whereas it decreases significantly at temperatures higher than 30–35 °C [42]. People eat more when exposed to cold than hot temperatures as the body needs more energy to maintain the temperature and increase in activity level [43]. During exposure to cold temperatures, an increase in the metabolic rate is observed in humans, which is also associated with an increase in caloric expenditure [44]. Therefore, the low sensitivity to tastes and low metabolic rate at high temperatures may be responsible for the small changes in the body weight and intestinal flora structure in mice at high temperatures.

## 5. Conclusions

In this study, we investigated the taste preferences of mice for different flavors of food (sweet, sour, bitter, and salty food) and the effects of these flavors on changes in the body weight and gut flora in mice. The results showed that mice have a strong preference for sweet and salty flavors, which is not affected by the temperature of the mice’s environment. Specific flavors in the diet, especially sour, sweet, and bitter led to significant changes in the abundance of specific microbial species in the microbiota, including *Akkermansia*, *Helicobacter*, *Bifidobacterium*, *Lachnospiraceae*, and *Bacteroides*. These differences were significantly influenced by ambient temperature. The continuous consumption of sour and sweet diets in cold environments resulted in changes in the functional pathways of the gut microbiota, including pathways related to carbohydrate metabolism. These changes may contribute to the observed changes in the body weight of the mice. Overall, we suggested that the combination of food flavors and different environmental temperatures leads to changes in the structure of the gut microbiota, thereby affecting metabolic function and body weight. Future research should focus on the specific mechanisms by which sour and sweet diets in cold environments alter the gut microbiota and metabolism and, in particular, investigate the changes in carbohydrate metabolism pathways and the interactions between microbial species. Additionally, the controlled laboratory conditions do not fully replicate natural living environments. Incorporating more natural settings and longer study durations would help to better understand the real-world implications of these findings.

## Figures and Tables

**Table 1:** The compositions of different flavors of feed (g/kg).

	Normal	Sweet	Salty	Sour	Bitter
Protein	178	178	178	178	178
Carbohydrate	643	628	633	642	643
Fat	70	70	70	70	70
Amino acid	1.7	1.7	1.7	1.7	1.7
Vitamin	4.8	4.8	4.8	4.8	4.8
Minerals	13.7	13.7	13.7	13.7	13.7
Moisture	66	66	66	66	66
Flavor additives	0	Sucrose 15	NaCl 10	Citric acid 1	Quinoline 0.008

## Data Availability

The original contributions presented in the study are included in the article. Further inquiries can be directed to the corresponding author.
